# Supplementary material for: Ionic Levothyroxine Formulations: Synthesis, Bioavailability, and Cytotoxicity Studies
Source: Int J Mol Sci. 2023 May 16;24(10):8822. doi: 10.3390/ijms24108822 (PMC10218257; doi:10.3390/ijms24108822)
Supplement: Supplementary file 1 [file ijms-24-08822-s001.zip › ijms-2362144-supplementary.pdf]

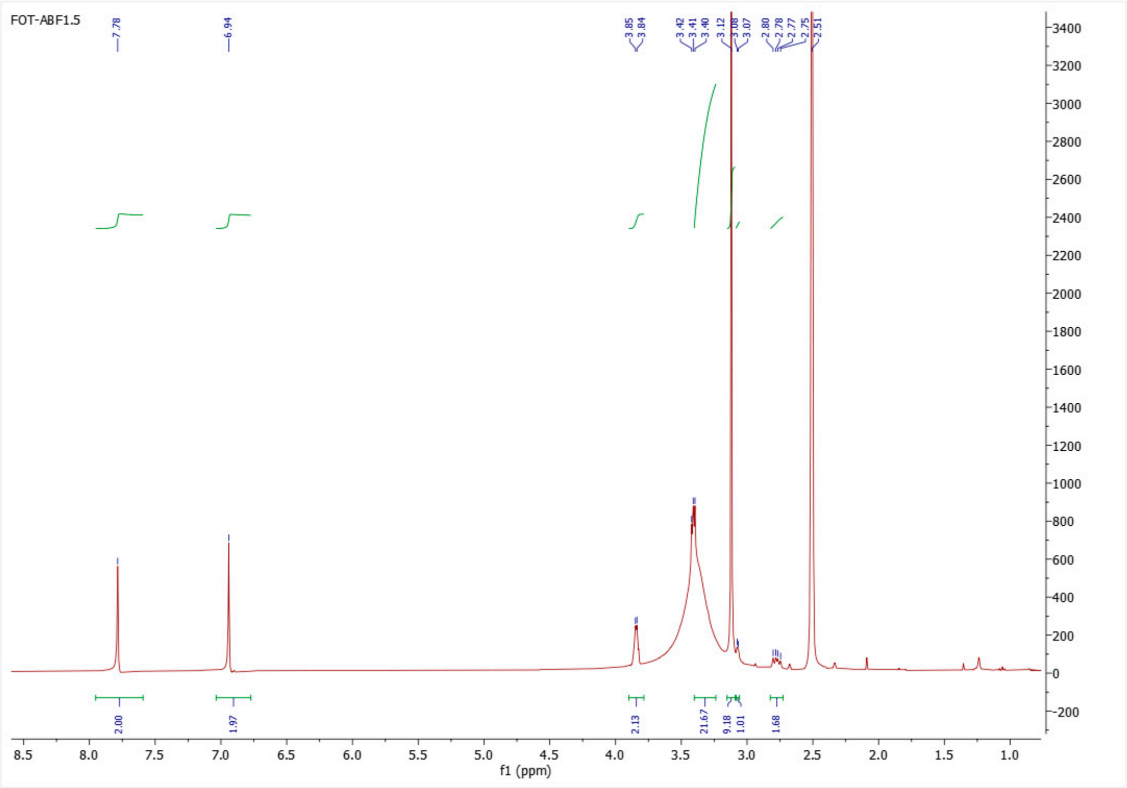

Figure S1 – ^{1}H-NMR of [Ch][T4]

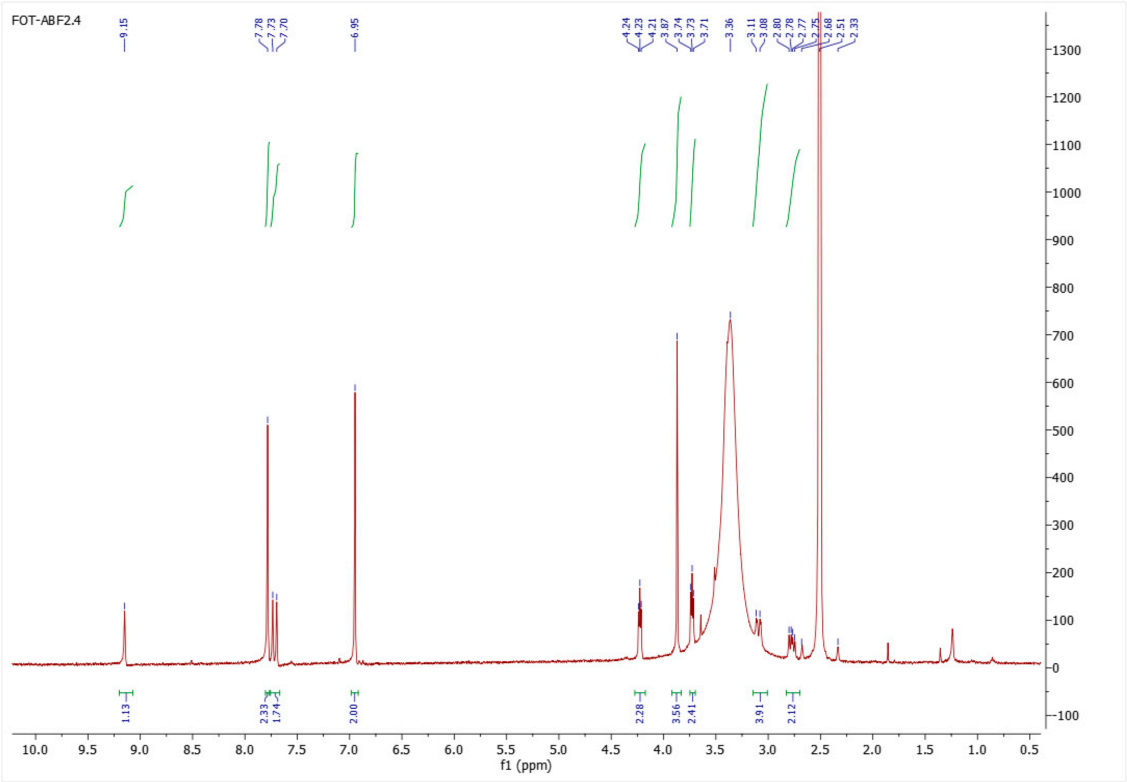

Figure S2 – ^{1}H-NMR of [C_2OHMiM][T4]

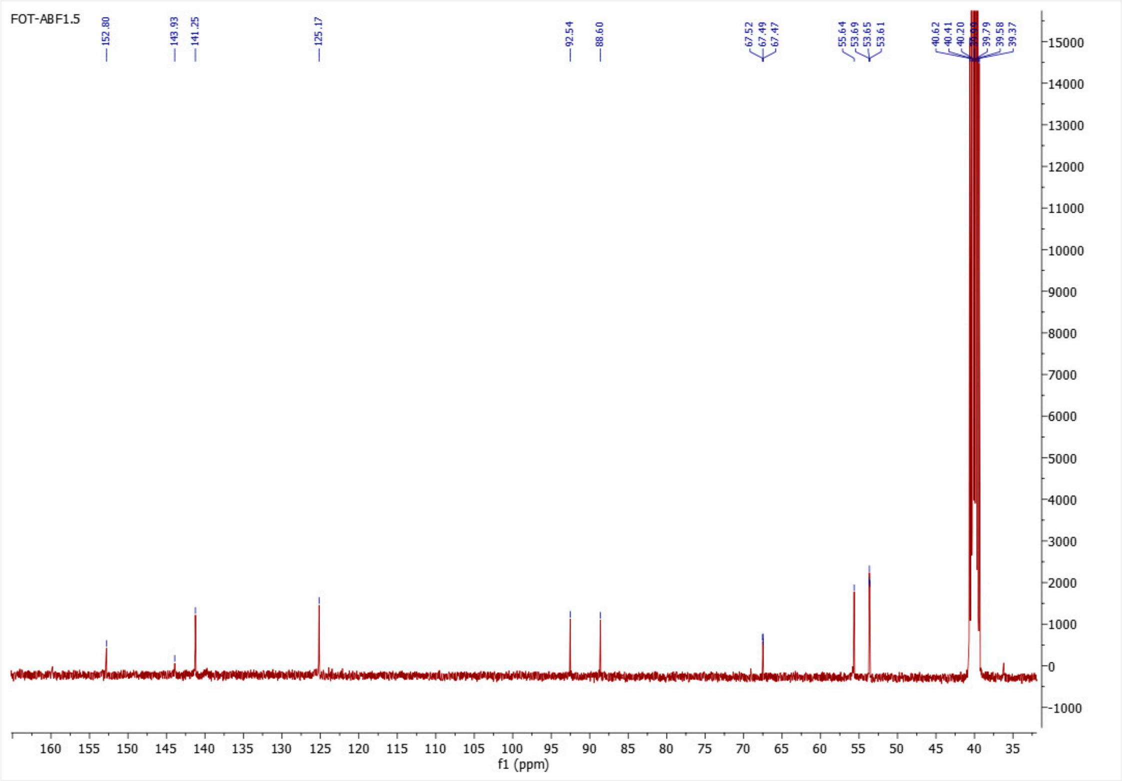

Figure S3 – ^{13}C-NMR of [Ch][T4]

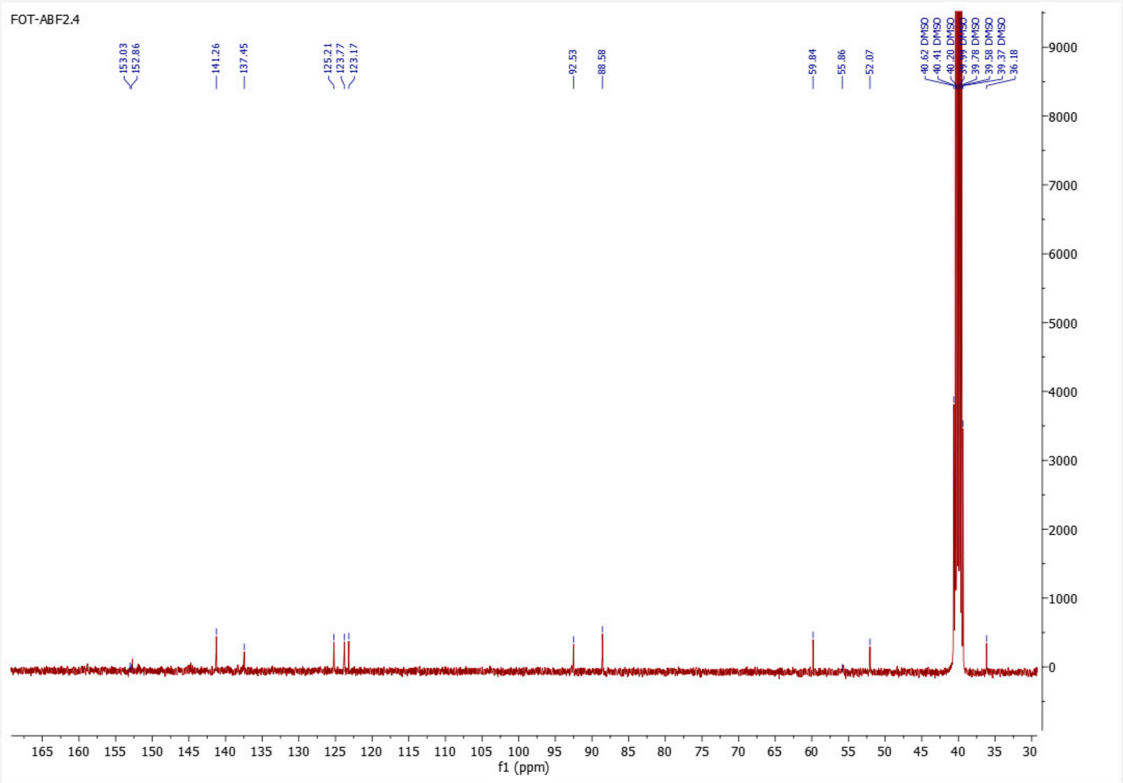

Figure S4 – ^{13}C-NMR of [C_2OHMiM][T4]

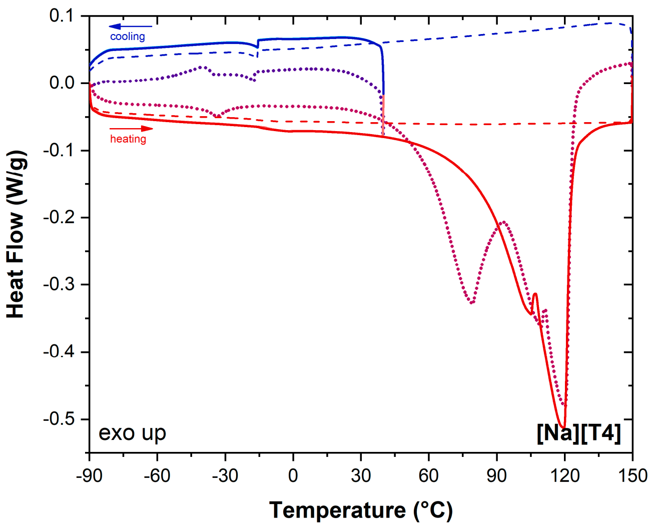

Figure S5 - Thermogram of neat levothyroxine, evidencing the effect of removing the adsorbed water by drying under vacuum prior to the calorimetric analyses (solid and dashed lines for the first and second runs, respectively). Dotted lines correspond to the DSC curves of [Na][T4] without any pre-treatment.

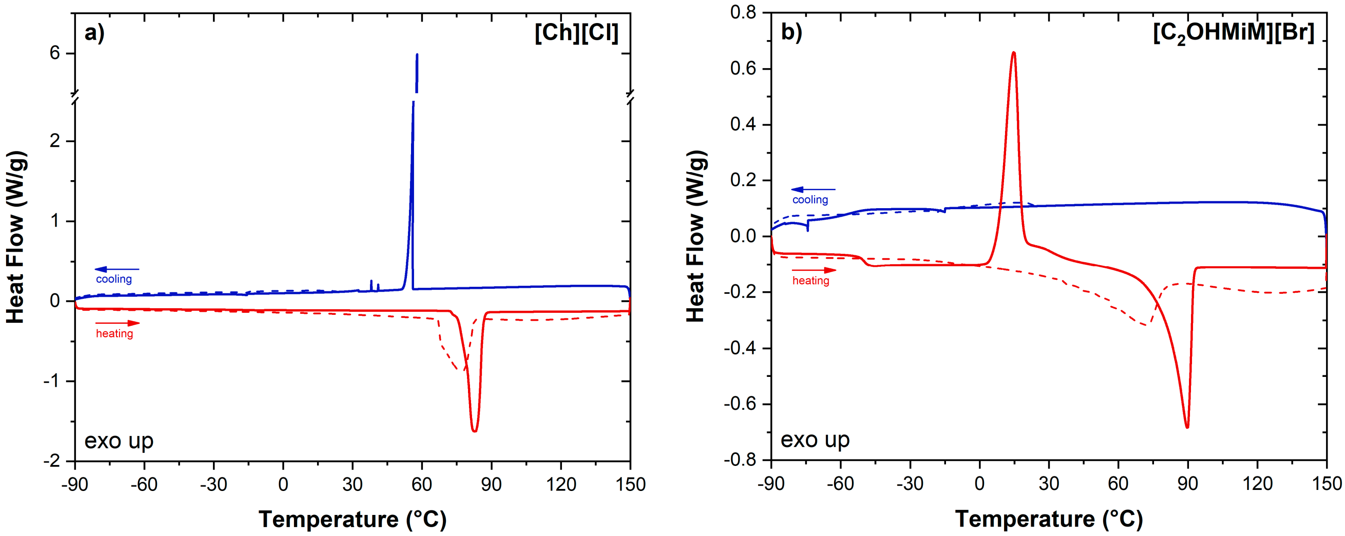

Figure S6 - Thermograms for the starting ionic liquids: a) [Ch][Cl] and b) [C2OHMiM][Br]. Dashed lines represent the first heating and cooling runs, whereas the solid lines correspond to the second heating and cooling runs

In the first heating run (red dashed line), [Ch][Cl] and [C2OHMiM][Br] exhibit simultaneously water removal and melting, while the following scans revealed several processes. For the first ionic liquid, crystallization (on cooling) and subsequent melting were found, whereas [C2OHMiM][Br] displayed vitrification, cold crystallization and the respective melting upon temperature increase.
